# *Paenibacillus terrisolis* sp. nov.: A Novel Strain Isolated from Heavy Metal Polluted Soil

**DOI:** 10.3390/microorganisms14051044

**Published:** 2026-05-05

**Authors:** Haoyu Wu, Congguo Ran, Supattra Kitikhun, Nan Zhou, Xingyu Liu, Chengying Jiang

**Affiliations:** 1 Institute of Earth Sciences, China University of Geosciences, Beijing 100083, China; 2009210013@email.cugb.edu.cn; 2 State Key Laboratory of Microbial Diversity and Innovative Utilization, Environmental Microbiology Research Center at Institute of Microbiology, Chinese Academy of Sciences, Beijing 100101, China; 3 University of Chinese Academy of Sciences, Beijing 100049, China; 4 Thailand Bioresource Research Center (TBRC), National Center for Genetic Engineering and Biotechnology, National Science and Technology Development Agency, Pathumthani 12120, Thailand

**Keywords:** *Paenibacillus terrisolis* sp. nov., *Paenibacillus*, *Paenibacillaceae*

## Abstract

A newly discovered facultative anaerobic strain, designated as LXY-3^T^, was obtained from a soil sample collected at an industrial site in Guangxi, China, known for heavy metal processing. An investigation including phenotypic, chemotaxonomic, and genomic traits was conducted. Phylogenetic analysis based on 16S rRNA showed that LXY-3^T^ belonged to the genus *Paenibacillus*. The closest phylogenetic relative of this strain was *Paenibacillus anaericanus* MH21^T^ with the similarity of 97.03%. Iso-C_15:0_, antéiso-C_15:0_, and C_16:1_ ω7c alcohol were the major cellular fatty acids. The predominant polar lipids comprised diphosphatidylglycerol (DPG), phosphatidylethanolamine (PE), phosphatidylglycerol (PG), unidentified phospholipids (PL1-PL8), unidentified resistant material (RM1–RM4), and lipids (L1–L3). For genome sequencing, the genomic DNA G+C content of the strain is 51.2 mol%. Comparative genomic analysis revealed that the average nucleotide identity (ANI) values between strain LXY-3^T^ and its closest phylogenetic relatives within the genus *Paenibacillus* (represented by type strains) were consistently below the 95% species demarcation threshold. Nitrogen fixation gene cluster (*nifB*, *nifE*, *nifK*, *nifN*, *nifV*, *nifX*, *nifD*, and *nifH*) was conserved in the strain. Correspondingly, digital DNA–DNA hybridization (dDDH) values remained below the 70% cutoff for species delineation. These genomic metrics provide compelling evidence that strain LXY-3^T^ represents a novel species within the genus *Paenibacillus*. The type strain LXY-3^T^ (=CGMCC 1.64949^T^ = JCM 37600^T^) is proposed.

## 1. Introduction

The genus *Paenibacillus* was first proposed by Ash et al. [1] based on the 16S rRNA gene sequences of group 3 bacilli [2] belonging to the family Paenibacillaceae in the order Caryophanales of the class Bacilli. As of now, there are 428 *Paenibacillus* species and 4 subspecies that have been validly published according to LPSN (https://lpsn.dsmz.de/search?word=Paenibacillus, accessed on 26 March 2026). Recent research endeavors have revealed that the members of this genus display a widespread ecological distribution, including soil [3], food [4], marine sediments [5], and animal guts [6]. Some species have been isolated from specific habitats, such as a paper mill [7], a necrotic wound [8], and blood cultures [9]. It has been reported that the genus *Paenibacillus* exhibits a variety of functional traits, encompassing nitrogen-fixing capabilities [10,11,12] and xylan hydrolysis [13,14]. Additionally, certain strains within this genus can serve as bioflocculants, facilitating the efficient harvesting of algal cells [15].

In recent years, the demand for various precious metal resources in industrial production and daily life has continued to rise. Simultaneously, heavy metal pollution has also increased, leading to serious harm for ecological security and human health. In the field of heavy metal pollution remediation, although traditional physicochemical methods can partially alleviate pollution, their high costs and potential risks of secondary pollution have restricted their large-scale application. In contrast, microbial remediation technology, with its notable advantages of low cost, environmental friendliness, and strong sustainability, has gradually become a research hotspot in this field. However, the effectiveness of microbial remediation technology hinges on the ability of suitable indigenous microorganisms. 

In this study, *Paenibacillus terrisolis* sp. nov., isolated from soil in a Pb-Zn smelting plant, a new species belonging to the genus *Paenibacillus* and designated as LXY-3^T^ was described. In the soil samples under investigation, the measured concentrations of heavy metals were as follows: lead (Pb) exhibited a content of 2269 mg/kg, zinc (Zn) reached 19,052 mg/kg, antimony (Sb) was present at 863 mg/kg, cadmium (Cd) recorded 244 mg/kg, and tin (Sn) showed a concentration of 810 mg/kg. Phylogenetic analysis revealed that it is closely related to the strain *Paenibacillus anaericanus* MH21^T^ [16] and *Paenibacillus segetis* DB 13260^T^ [17]. This taxonomic proposal is supported by thorough analyses of the genotypic, chemotaxonomic, and phenotypic traits. Due to the chronic contamination with high concentrations of toxic metals in the soil sample, the genome of LXY-3^T^ was revealed as metal resistance genes coupled with a complete nitrogen fixation gene pathway, which expressed a significant role in heavy metal contaminated sites.

Its ability to persist under such harsh conditions can be attributed to microbes that likely evolved their specialized resistance mechanisms for living [18]. The majority of the *Paenibacillus* taxa exhibit non-diazotrophic characteristics, with nitrogen-fixing capacity restricted to merely several dozen species within this genus [19]. Notably, genomic analysis of LXY-3^T^ revealed the presence of key nitrogen fixation genes (*nifDKH*), which encode the structural subunits of the molybdenum-dependent nitrogenase complex [20]. This genetic evidence was further corroborated by phenotypic assays, confirming its functional role in biological nitrogen fixation.

Discovery of LXY-3^T^ expands our understanding of microbial adaptations to anthropogenic pollution. This not only serves as a promising microbial resource for the bioremediation and ecological restoration of polluted soils, but also offers insights into the molecular mechanisms underlying stress resistance and metabolic versatility. Further characterization of LXY-3^T^’s resistance determinants (e.g., metal-binding proteins and efflux pumps) and nitrogenase regulation under stress conditions can have an advantage in terms of engineered microbes for sustainable environmental management. Collectively, these findings underscore the ecological and biotechnological importance of exploring microbial diversity in polluted industrial niches.

## 2. Materials and Methods

### 2.1. Isolation and Culturing

Soil specimens were retrieved from an industrial facility specializing in heavy metal smelting, located in Guangxi Province, China (23°42′2″ N, 109°12′10″ E). A quantity of approximately 20 g of soil sample was dispensed into a 50 ml centrifuge tube by aseptic technique and maintained at 4 °C during transportation to the laboratory to ensure sample integrity.

For the preparation of samples, a 5 g portion of soil was introduced into 45 mL of sterile water within a centrifuge tube and shaken vigorously for 10 min to ensure a uniform suspension of soil particles. After allowing 10 min for sedimentation to facilitate the separation of solid elements, the supernatant was gathered and employed as the bacterial suspension for the ensuing screening steps. This bacterial suspension underwent a sequence of 10-fold progressive dilutions, spanning from 10^−1^ to 10^−7^ [18]. The R2A medium showed broad cultivation of diverse environmental microorganisms across broad phylogenetic lineages, encompassing species characterized by sluggish growth kinetics and a preference for oligotrophic nutritional conditions [21]. Subsequently, each diluted sample was evenly distributed onto R2A agar plates and cultivated at 30 °C for a duration of 7 days.

A total of 100 microbial isolates were acquired and subsequently purified through the streak-plating method. Among these isolates, strain LXY-3^T^ was identified as a potential novel species through a comparative analysis of its 16S rRNA gene sequence against the EzBioCloud database (https://www.ezbiocloud.net/, accessed on 26 March 2026) [22,23]. For long-term preservation, strain LXY-3^T^ was stored as a 50% (v/v) glycerol suspension at −80 °C. 

### 2.2. Morphological and Physiological Analysis

Phenotypic traits, biochemical features, as well as the composition of polar lipids and cellular fatty acids, were assessed under conditions favorable for optimal growth. Cell morphology was inspected via scanning electron microscopy (SU8010, Hitachi, Tokyo, Japan), flagella were visualized using a transmission electron microscope (TEM, JEM-1400, JEOL, Akishima, Japan), and cell motility was determined through light microscopy (Axiostar Plus, ZEISS, Oberkochen, Germany). Gram staining was conducted following 72 h of incubation at 30°C, strictly adhering to the instructions provided in the commercial Gram staining kit protocol (Catalog No. G1060, Solarbio, Beijing, China) [24]. The growth temperature range was investigated at 4, 22, 30, 37, 42, 50, and 60 °C for 72 h, while the growth pH range was determined in R2A broth adjusted to 4-12 at intervals of 1.0, with incubation at 30 °C for 72 h. Salt tolerance was tested in R2A broth supplemented with 0–5% (w/v) NaCl in 1% intervals. Cell growth was measured by determining the optical density at 600 nm (OD_600_) using a UV/Vis spectrophotometer (SPECORD 205; Analytik Jena, Jena, Germany). Biochemical and enzymatic properties were analyzed utilizing GEN III microplates (Biolog, Hayward, CA, USA), the API 20 NE system, and the API ZYM system (bioMérieux, Marcy-l’Étoile, France), adhering to the manufacturers’ instructions [25]. Antibiotic susceptibility was evaluated via the single-disk diffusion method [26], with inhibition zone diameters serving as sensitivity indicators. The composition of whole-cell fatty acids and polar lipid profiles was determined based on previously established methodologies [27].

### 2.3. 16S rRNA Gene Sequencing and Phylogenetic Analysis

The 16S rRNA gene of the pure culture was amplified by using universal primers 27F (5′-AGAGTTTGATCCTGG CTCAG3′) and 1492R (5′-GGTTACCTTGTTACGACTT-3′), under the following conditions: 95 °C for 5 min; 30 cycles of 95 °C for 30 s, 55 °C for 30 s, 72 °C for 1.5 min; and final extension at 72 °C for 10 min [28]. The amplified PCR products underwent Sanger sequencing at Beijing Tianyihuiyuan Biotechnology Co., Ltd. (Beijing, China) [29]. Bacterial species identification was achieved through comparative analysis of the 16S rRNA gene sequences against reference strains in the EzBioCloud database (https://www.ezbiocloud.net/; accessed 26 March 2026). Subsequently, phylogenetic relationships were reconstructed using the neighbor-joining (NJ) algorithm [30], in accordance with Kimura’s two-parameter model [31], utilizing MEGA software (version 7.0). Additionally, the maximum-likelihood (ML) method [32] based on the Tamura–Nei model, as well as the maximum-parsimony (MP) method [33], were also applied for tree reconstruction. The nodal support levels were assessed through 1000 bootstrap replicates for both NJ and ML analyses, and 500 replicates for the MP analysis (refer to Figures S2 and S3) [34].

### 2.4. Genome Sequencing and Analysis

Genomic DNA was extracted in accordance with established methodologies [18]. Sequence quality control was conducted utilizing fastp (v0.20.0), whereas gene prediction and coding sequence (CDS) identification were executed employing Prodigal (v2.6.3) and GeneMarkS (v4.3), respectively. The raw sequencing data underwent a thorough quality evaluation [35], followed by trimming and de novo assembly using SOAPdenovo (v2.04) [36]. To evaluate the phylogenomic placement and evolutionary connections among the strains, average nucleotide identity (ANI) and digital DNA–DNA hybridization (dDDH) values were calculated between the bacterial strains in question and their closest phylogenetic relatives. dDDH values were computed via the web-based Genome-to-Genome Distance Calculator (GGDC, v2.1; http://ggdc.dsmz.de/ggdc.php, accessed on 26 March 2026) [37], whereas ANI was determined using the online ANI calculator (available at ANI Calculator|EzBioCloud.net) (https://www.ezbiocloud.net/tools/ani, accessed on 26 March 2026).

## 3. Results and Discussion

### 3.1. Morphological Genotypic and Physiological Investigation

**Figure 1:**
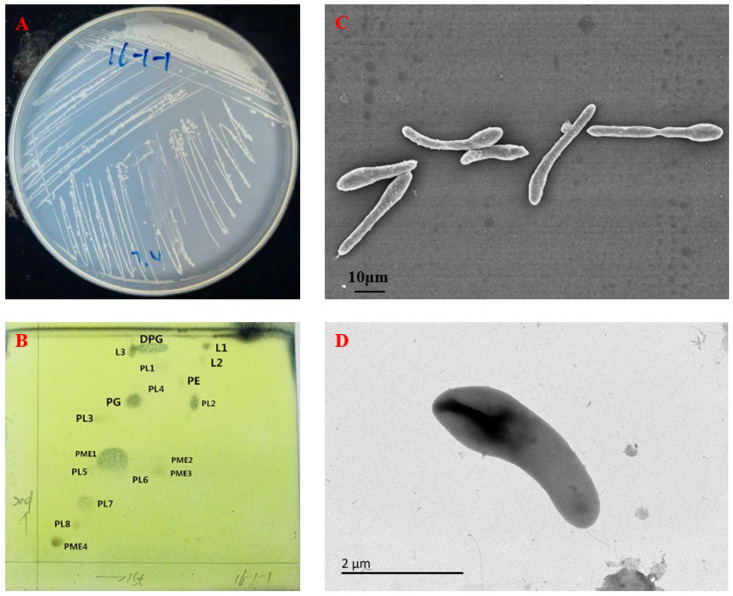
Cell and colony morphology of strain LXY-3^T^. (**A**) Colony morphology after three days of cultivation on R2A agar plates for strain LXY-3^T^; (**B**) polar lipids profiles of strain LXY-3^T^. DPG: diphosphatidylglycerol; PE: phosphatidylethanolamine; PG: phosphatidylglycerol; PL: phospholipid; (**C**) transmission electron micrograph of LXY-3; (**D**) scanning electron micrograph of LXY-3^T^.

After 3 days’ cultivation on R2A solid medium, the colonies of strain LXY-3^T^ were white, opaque, circular, flat, and smooth, with a moist surface, 0.5–1 mm in diameter, and with slightly raised surfaces (Figure 1A). Transmission electron microscopy revealed that cells were spindle-shaped, with widths ranging from 0.8 to 1.2 µm and variable lengths between 2.8 and 32.6 µm (Figure 1C). Flagella can be observed from the scanning electron microscope photograph (Figure 1D). Compared with the reference strain *Paenibacillus anaericanus* MH21^T^, strain LXY-3^T^ differed from the reference strain *Paenibacillus anaericanus* MH21^T^ which exhibits a rod-shaped cell form, whereas LXY-3^T^ displays a distinct spindle-shaped morphology. The morphological distinctions suggest potential taxonomic differentiation between the two strains. Gram staining confirmed that strain LXY-3^T^ was Gram-negative. The Gram staining variability among *Paenibacillus* species (Table 1) reflects differences in cell wall structure, particularly the thickness and cross-linking of peptidoglycan layers. For example, *P. anaericanus* MH21^T^ (Gram-negative) and *P. segetis* DB 13260^T^ (Gram-positive) exhibit distinct cell wall compositions, consistent with their phylogenetic divergence.

Strain LXY-3^T^ exhibited a broad temperature growth range from 22 °C to 50 °C, achieving its optimal growth performance at 30 °C. Its pH tolerance spanned from 5 to 11, with the most favorable growth occurring at a pH of 9. Notably, LXY-3^T^ was completely intolerant to NaCl (Figure S1).

In contrast, the reference strain *Paenibacillus anaericanus* MH21^T^ had an optimal growth at 30 °C to 35 °C and a pH 7.7. These comparative analyses clearly highlight the significant phenotypic differences in the growth profiles between strain LXY-3^T^ and *Paenibacillus anaericanus* MH21^T^ (Table 1).

Strain LXY-3^T^ was streaked onto nitrogen-free Ashby solid medium [38] and incubated for three days. During this period, a distinct phenotypic alteration was observed: the solid medium adjacent to the bacterial colonies transitioned from an opaque white appearance to a transparent state, forming clear halos around the colonies. This phenomenon resulted from the metabolic activity of strain LXY-3^T^, which produced organic acids during nitrogen fixation. These acids diffused into the surrounding medium and reacted with calcium carbonate, leading to its dissolution via acid–carbonate neutralization reactions (CaCO_3_ + 2H^+^ → Ca^2+^ + CO_2_↑ + H_2_O). Such a phenotypic manifestation suggests that strain LXY-3^T^ possesses nitrogen-fixing capabilities, as the formation of transparent zones in Ashby medium is a classic indicator of biological nitrogen fixation mediated by acid production.

The substrate utilization capabilities of strain LXY-3^T^ were systematically analyzed using GEN III MicroPlates (Biolog) (Biolog, Hayward, CA, USA), API ZYM, and API 20 NE systems (bioMérieux, Marcy-l’Étoile, France). LXY-3^T^ could metabolize common substrates including Dextrin, D-Maltose, D-Trehalose, D-Cellobiose, Gentiobiose, D-Turanose, Stachyose, *α*-D-Lactose, D-Melibiose, D-Salicin, N-Acetyl-D-Glucosamine, N-Acetyl-*β*-D-Mannosamine, *α*-D-Glucose, D-Mannose, D-Galactose, D-Mannitol, and D-Gluconic acid (Table S1). It exhibited a narrower substrate spectrum compared with *Paenibacillus anaericanus* MH21^T^. Moreover, *Paenibacillus anaericanus* MH21^T^ can grow aerobically on saccharose, starch, chitin, and inulin, which cannot be utilized by LXY-3^T^.

Enzymatic activity assays (API ZYM and API 20 NE systems) showed that LXY-3^T^ exhibited activities for Esterase (C4), Esterase (C8), Phosphoamidase, *α*-Galactosidase, *β*-Galactosidase, *β*-Galactosidase, *β*-glucosidase, and Cytochrome oxidase (Tables S2 and S3).

**Figure 2:**
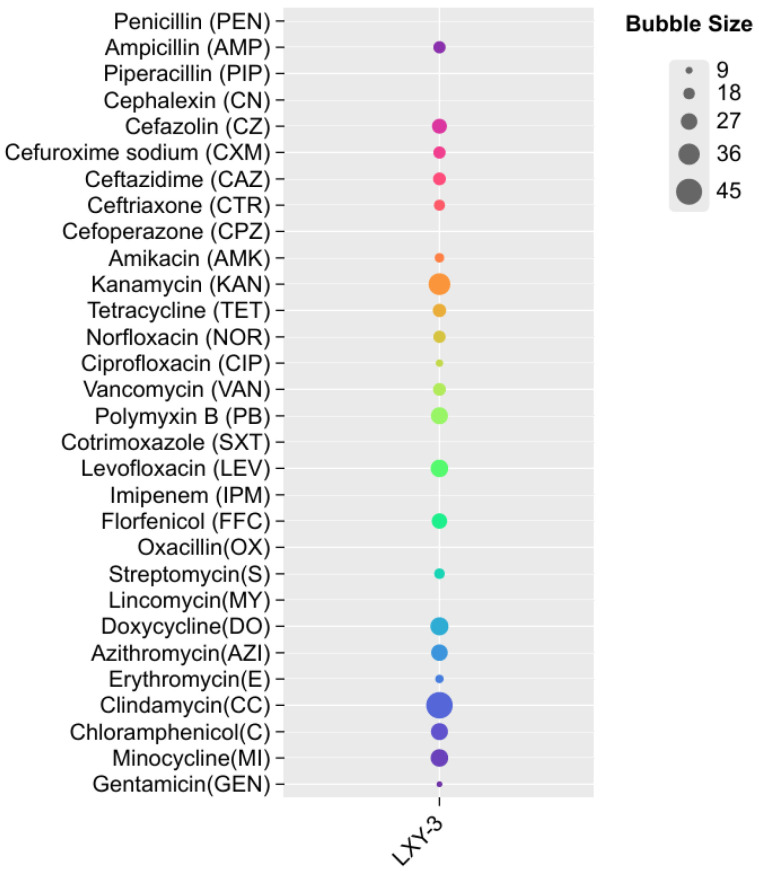
Antibiotic susceptibility profiles of strain LXY-3^T^, with bubble dimensions corresponding to the measured inhibition zone diameters (mm).

The antibiotic susceptibility profile of strain LXY-3^T^ demonstrated unique resistance characteristics, as illustrated in Figure 2. Notably, LXY-3^T^ was resistant to *β*-lactam antibiotics, encompassing cefazolin, cefuroxime sodium, ceftazidime, and ceftriaxone. Conversely, it displayed moderate susceptibility to aminoglycosides, including gentamicin and streptomycin. No susceptibility was observed to penicillin, piperacillin, cephalexin, cefoperazone, cotrimoxazole, imipenem, oxacillin, and lincomycin. These phenotypic traits not only provide significant taxonomic markers but also potentially reflect distinct ecological adaptations linked to the resistance profiles of individual strains.

### 3.2. Phylogenetic Analysis

**Figure 3:**
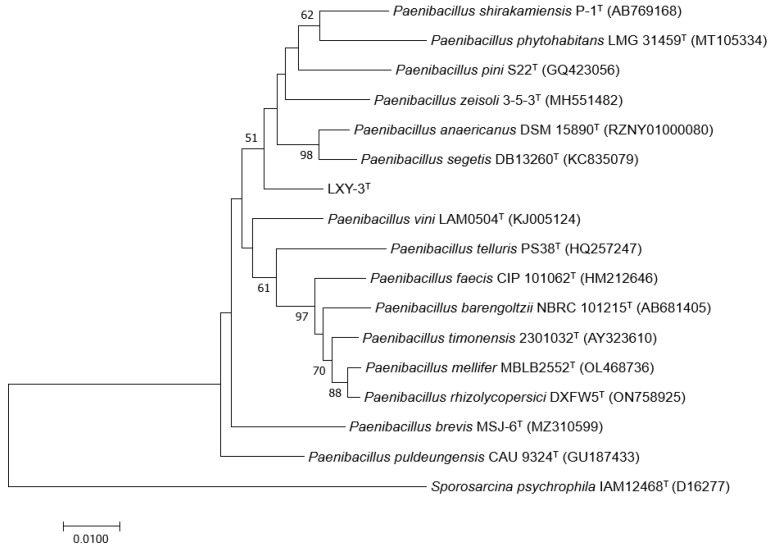
Maximum-likelihood phylogenetic reconstruction of strain LXY-3^T^ and its phylogenetically affiliated type strains within the genus *Paenibacillus*. The neighbor-joining algorithm was employed for tree construction using 16S rRNA gene sequences. *Sporosarcina psychrophila* IAM12468^T^ was designated as the outgroup taxon. All nucleotide sequences were obtained from the GenBank database, with corresponding accession numbers listed in parentheses. Branch nodes display bootstrap support values (≥50% from 1000 replicates) indicating clade confidence levels. The horizontal scale bar corresponds to 0.01 nucleotide substitutions per site. The final alignment comprised 1399 conserved positions after trimming ambiguous regions.

The 16S rRNA gene sequences of strain *Paenibacillus terrisolis* LXY-3^T^ have been deposited under the accession number PRJNA1185323. Phylogenetic analysis of 16S rRNA gene sequences indicated that the closest relative of strain LXY-3^T^ was the type strain *Paenibacillus anaericanus* MH21^T^ (with 97.03% identity) [6], followed by *Paenibacillus puldeungensis* CAU 9324^T^ (96.85%) [16] and *Paenibacillus segetis* DB 13260^T^ (96.59%) [17] (Figure 3).

### 3.3. Genome Characteristics

**Figure 4:**
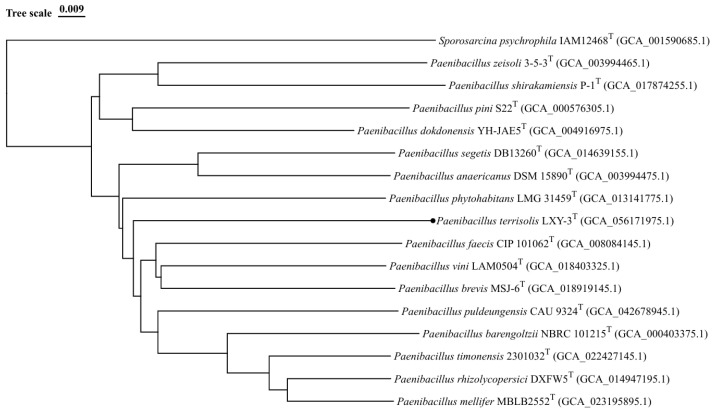
Phylogenomic tree of strain LXY-3^T^ within the members of genus *Paenibacillus*. Phylogenetic relationships at the genomic level of strain LXY-3^T^ and the species of the genus *Paenibacillus*. GenBank accession numbers of the genomes used are given in parentheses. The gene support indices indicate the number of single gene trees supporting each branch in the tree from the concatenated alignment and are marked on the branches. *Sporosarcina psychrophila* IAM12468^T^ was used as the out-group. Bars, 0.009 substitution per site.

Phylogenomic reconstruction based on CVTree 3.0 (https://cvtree.online/v3/cvtree/, accessed on 26 March 2026) robustly placed strain LXY-3^T^ within the genus *Paenibacillus*, with each strain forming a well-supported monophyletic clade (Figure 4) [39]. Notably, strain LXY-3^T^ showed relatedness to *Paenibacillus faecis* CIP 101062^T^, *Paenibacillus vini* LAM0504^T^, and *Paenibacillus brevis* MSJ-6^T^.

The whole-genome sequence of strain LXY-3^T^ (GenBank accession no. JBKFGG000000000) is 5.36 Mb in length with a GC content of 51.23 mol%. Based on the KEGG annotation, it revealed that the genome of LXY-3^T^ encodes genes associated with various biological processes, including cellular processes (283 genes), metabolism (4294 genes), genetic information processing (272 genes), organismal systems (75 genes), human diseases (222 genes), and environmental information processing (616 genes) (Table S4).

**Figure 5:**
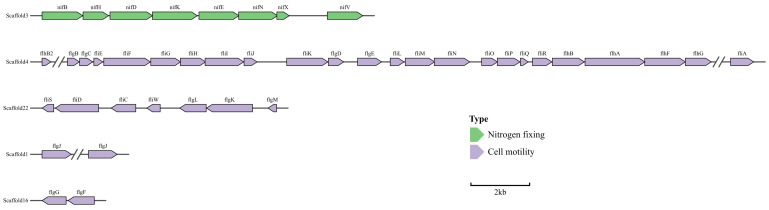
Nitrogen fixing genes and cell motility genes of strain LXY-3^T^.

Notably, the genome of LXY-3^T^ has been annotated to include genes closely associated with flagellar assembly and motility. These genes encompass a comprehensive array of components, including basal body forming, hook, filament, and regulatory elements, all of which synergistically relate to their flagellum biogenesis and functions. The identified genetic determinants specifically encode key structures such as the MS/C ring, Type III secretion system (T3SS), rod/hook elements, filament proteins, and a suite of regulatory factors, presenting a holistic genomic framework of flagellar motility (Figure 5). This genomic revelation is robustly corroborated by phenotypic observations, confirming the functional translation of flagellar genes into active motility phenotypes. The possession of a fully operational flagellar apparatus not only endows the bacterium with enhanced mobility but also plays a pivotal role in its adaptive strategies within diverse environmental milieus, such as facilitating chemotactic responses toward optimal niches or enabling evasion in various conditions. Also, genomic annotation has revealed a set of genes implicated in heavy metal resistance, including *arsA*, *arsB*, *arsC*, and *arsR* [40]. Additional determinants such as *katE*, *ZntA*, and *ZntR* further underscore the strain’s capacity to tolerate metal toxicity [41]. These findings suggest that LXY-3^T^ employs a multifaceted strategy to counteract environmental stressors, integrating motility-driven niche exploration with metal resistance mechanisms, which is a trait advantageous in metal-contaminated habitats.

Similar to other strains in the genus *Paenibacillus*, genomic annotation revealed that strain LXY-3^T^, like some other members of the genus *Paenibacillus* [42], possess KEGG-annotated genes involved in nitrogen cycle processes. The strain LXY-3^T^ revealed the presence of nitrogen cycle-associated genes, specifically *nifDKH* (Figure 5). These genetic elements are well-established as conferring the ability to fix atmospheric nitrogen, converting it into forms accessible to the organism. The concordance between the observed phenotypic evidence and the genomic predictions provides robust validation for the functional expression of nitrogen fixation pathways in strain LXY-3^T^.

Therefore, the nitrogen-fixing ability of strain LXY-3^T^ holds significant ecological implications. In terms of biogeochemistry, nitrogen fixation represents a critical process that replenishes bioavailable nitrogen in nitrogen-limited ecosystems, thereby sustaining autotrophic carbon fixation and biogeochemical element cycling. Nitrogen-fixing microorganisms play a main role in maintaining ecosystem resilience and soil fertility. The demonstration of nitrogen fixation in strain LXY-3^T^ not only enhances our understanding of its ecological functions but also underscores its potential as a biofertilizer candidate for promoting plant growth in nitrogen-deficient soils, thereby contributing to agroecosystem sustainability.

The co-occurrence of heavy metal resistance and nitrogen-fixing capabilities in LXY-3^T^ showed a significant relationship between isolation source and strain characteristics. In contaminated industrial soils, heavy metal toxicity often disrupts microbial nitrogen cycling by inhibiting nitrifying and denitrifying communities, leading to nitrogen imbalance and reduced soil fertility [43]. The ability of LXY-3^T^ to fix atmospheric nitrogen (N_2_) into bioavailable ammonia (NH_3_) under metal stress suggests its potential to counteract nitrogen depletion in such degraded environments [44]. This dual functionality, including heavy metal resistance coupled with nitrogen fixation, positions LXY-3^T^ as a promising candidate for bioremediation applications. By reducing heavy metal bioavailability through adsorption or transformation while simultaneously enhancing soil nitrogen content, this strain could facilitate ecosystem restoration in contaminated sites [45].

**Figure 6:**
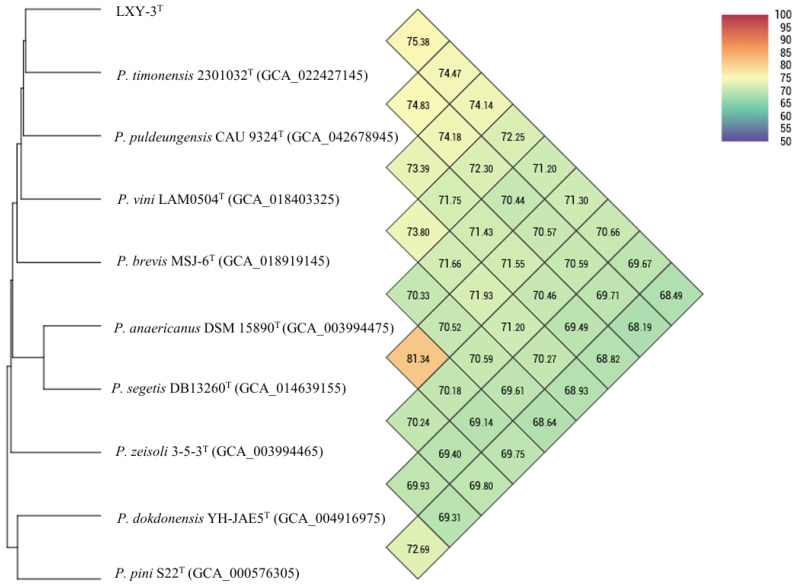
ANI heatmap of strain LXY-3^T^, along with their closely related strains.

Comprehensive genomic analyses reveal that the novel strain LXY-3^T^ displays taxonomic and functional traits that substantiate its classification as a distinct species within the genus *Paenibacillus*. The G+C content of LXY-3^T^ (51.23 mol%) falls within the range reported for *Paenibacillus* species (39–59 mol%) [46,47], but differs from closely related strains such as *P. anaericanus* MH21^T^ (42.6 mol%) and *P. segetis* DB 13260^T^ (53.7 mol%). This variability reflects genus-wide diversity in nucleotide composition, likely driven by horizontal gene transfer or adaptive evolution. Moreover, phylogenomic and comparative genomic analyses offer compelling evidence that support its novel taxonomic status. Specifically, the dDDH value with the closest relative is 19.50%, and the highest ANI value is 75.38%, between LXY-3^T^ and *Paenibacillus puldeungensis* CAU 9324^T^. Both values are significantly below the established thresholds for species demarcation (dDDH < 70%, ANI < 95%) (Figure 6). These molecular boundaries, coupled with its unique genomic features, collectively satisfy the polyphasic criteria for proposing a novel species. The genome sequence accession number for LXY-3^T^ is provided in Data Availability Statement.

### 3.4. Chemotaxonomic Characterization

The predominant cellular fatty acids (≥10%) for strain LXY-3^T^ were iso-C_15:0_, antéiso-C_15:0_, and C_16:1_ *ω*7*c* alcohol, which is broadly consistent with patterns of other strains in the genus *Paenibacillus*. LXY-3^T^ has iso-C_17:0_ like the type strain *Paenibacillus anaericanus* MH21^T^ and *Paenibacillus segetis* DB 13260^T^, but unlike the strain *Paenibacillus puldeungensis* CAU 9324^T^. However, comparing with the three type strains in Table 2, LXY-3^T^ has a lower proportion of C_16:0_ compared to the three reference type strains. Detailed information on cellular fatty acids components is shown in Table 2.

The profile contained the major phospholipids diphosphatidylglycerol (DPG), phosphatidylethanolamine (PE), and phosphatidylglycerol (PG). Additionally, several unidentified components were detected, including phospholipids (PL1–PL8), resistant materials (RM1–RM4), and lipids (L1–L3).

Polar lipid distributions provided additional taxonomic discrimination (Figure 1B). Except the major polar lipids diphosphatidylglycerol (DPG), phosphatidylethanolamine (PE), and phosphatidylglycerol (PG), strain LXY-3^T^ contained several unidentified components, including phospholipids (PL1-PL8), resistant material (RM1-RM4), and lipids (L1-L3).

## 4. Conclusions

Based on phylogenetic and genomic analyses, strain LXY-3^T^ forms a distinct lineage within the genus *Paenibacillus*, supported by a 16S rRNA gene sequence similarity below 98.5%, an ANI value below 95%, and a dDDH value below 70% compared to its closest relatives. These metrics collectively confirm its classification as a novel species within the genus. Functional characterization revealed that LXY-3^T^ harbors a complete nitrogen fixation gene cluster (including *nifB*, *nifE*, *nifK*, *nifN*, *nifV*, *nifX*, *nifD*, and *nifH*), enabling its participation in the nitrogen cycle through biological nitrogen fixation. This genetic feature distinguishes LXY-3^T^ from other *Paenibacillus* species and underscores its potential ecological role in nitrogen cycling within its native environment.

## Figures and Tables

**Table 1:** Multidimensional comparison of strain LXY-3^T^ with *Paenibacillus* type strains. Strains: 1, LXY-3^T^ (data from this study); 2, *Paenibacillus anaericanus* MH21^T^ (data from reference [6]); 3, *Paenibacillus puldeungensis* CAU 9324^T^ (data from reference [16]); 4, *Paenibacillus segetis* DB 13260^T^ (data from reference [17]); +, positive; −, negative; ANI stands for average nucleotide identity.

Characteristics	1	2	3	4
Source of isolation	Soil	Gut	Sandbank	Soil
Cell shape	Spindle-shaped	Rod-shaped	Rod-shaped	Rod-shaped
Anaerobic growth	+	+	+	+
Temperature (°C) Optimal (°C)	22–50 (30)	5–40 (30–35)	20–45 (30)	5–40 (30–37)
pH	5.0–11.0	5.8–8.6	5.0–11.0	6.0–10.5
Gram staining	−	−	+	+
Major cellular fatty acids	iso-C_15:0_	Antéiso C_15:0_	Antéiso C_15:0_	Antéiso C_15:0_
**Genome features**				
Genome size (Mb)	5.36	5.87	5.35	5.32
Mol% G+C	51.23	42.6	48.8	53.7
ANI	75.38	81.34	71.43	70.24
**Acid production from**				
Glucose	+	+	+	−
Fructose	−	−	−	−
D-Mannose	+	+	−	+
L-Arabinose	−	−	+	−
D-Galactose	+	−	−	+
Trehalose	+	−	−	+

**Table 2:** Cellular fatty acid profiles (% of totals) of strain LXY-3^T^, using data from this study; *Paenibacillus anaericanus* MH21^T^, *Paenibacillus puldeungensis* CAU 9324^T^, and *Paenibacillus segetis* DB 13260^T^, using data from reference [6,16,17]. Only fatty acids accounting for at least 1.0% of the total fatty acid content are listed.

Fatty Acid	Percentage (%)			
	LXY-3^T^	*Paenibacillus* *anaericanus* MH21^T^	*Paenibacillus puldeungensis* CAU 9324^T^	*Paenibacillus segetis* DB 13260^T^
C_12:0_	-	1.1	-	1.8
antéiso-C_13:0_	-	0.5	-	-
iso-C_14:0_	9.71	4.3	5.2	7.1
C_14:0_	1.08	14.5	3.0	3.0
iso-C_15:0_	35.66	5.5	3.9	5.9
antéiso-C_15:0_	18.74	35.6	53.2	43.9
C_15:0_	-	-	1.5	-
C_16:1_ *ω7c* alcohol	10.99	-	-	-
iso-C_16:0_	5.32	8.3	9.0	19.6
C_16:1_ *ω11c*	4.20	-	-	-
C_16:0_	2.57	26.3	17.0	13.5
iso-C_17:1_*ω10c*	2.66	-	-	-
iso-C_17:0_	1.05	1.4	-	1.6
antéiso-C_17:0_	3.51	1.9	3.9	2.3

## Data Availability

The 16S rRNA gene sequence of strain *Paenibacillus terrisolis sp. nov.* is PRJNA1185323. The whole-genome shotgun projects for *Paenibacillus terrisolis sp. nov.* has been deposited at DDBJ/ENA/GenBank under the accession number JBKFGG000000000. The respective version described in this paper is JBKFGG000000000.1.

## Supplementary Materials

The following supporting information can be downloaded at https://www.mdpi.com/article/10.3390/microorganisms14051044/s1, Figure S1. Growth dynamics of strain LXY-3^T^ under diverse environmental conditions. (a) Growth curves (OD_600_) of LXY-3^T^ cultured at different pH levels (4–12) at 30°C with 0% NaCl. (b) Growth profiles of LXY-3^T^ across a temperature gradient (4–50°C) at pH 7.0 with 0% NaCl. (c) Cell density (OD_600_) of LXY-3^T^ in media supplemented with increasing NaCl concentrations (0–5%) at 30°C and pH 7.0. Figure S2: Maximum-likelihood tree based on 16S rRNA gene sequences showing the phylogenetic position of strain LXY-3^T^ among the members of genus *Paenibacillus*. *Sporosarcina psychrophila* IAM12468^T^ served as the out-group. The sequences were obtained from GenBank. Bar, 0.02 substitutions per nucleotide position. The average size of the aligned and trimmed sequences used for phylogenetic tree construction is 1399 bp. Figure S3. Maximum-parsimony tree based on 16S rRNA gene sequences showing the phylogenetic position of strain LXY-3^T^ among the members of genus *Paenibacillus*. *Sporosarcina psychrophila* IAM12468^T^ served as the out-group. The sequences were obtained from GenBank. The average size of the aligned and trimmed sequences used for phylogenetic tree construction is 1399 bp. Table S1. Phenotypic characteristics of strain LXY-3^T^ determined by Biolog GEN III MicroPlate^TM^. Table S2. Phenotypic characteristics of strain LXY-3^T^ determined by the API ZYM kit. Table S3. Phenotypic characteristics of strain LXY-3^T^ determined by the API 20NE test kit. Table S4. Genome analysis of strain LXY-3^T^ in KEGG database.

## Abbreviations

The following abbreviations are used in this manuscript:

ANI	Average nucleotide identity
dDDH	Digital DNA–DNA hybridization
ML	Maximum-likelihood
MP	Maximum-parsimony
NJ	Neighbor-joining
R2A	Reasoner’s 2A
