# Supplementary material for: Paenibacillus terrisolis sp. nov.: A Novel Strain Isolated from Heavy Metal Polluted Soil
Source: Microorganisms. 2026 May 5;14(5):1044. doi: 10.3390/microorganisms14051044 (PMC13209447; doi:10.3390/microorganisms14051044)
Supplement: Supplementary file 1 [file microorganisms-14-01044-s001.zip › microorganisms-4275217-supplementary.pdf]

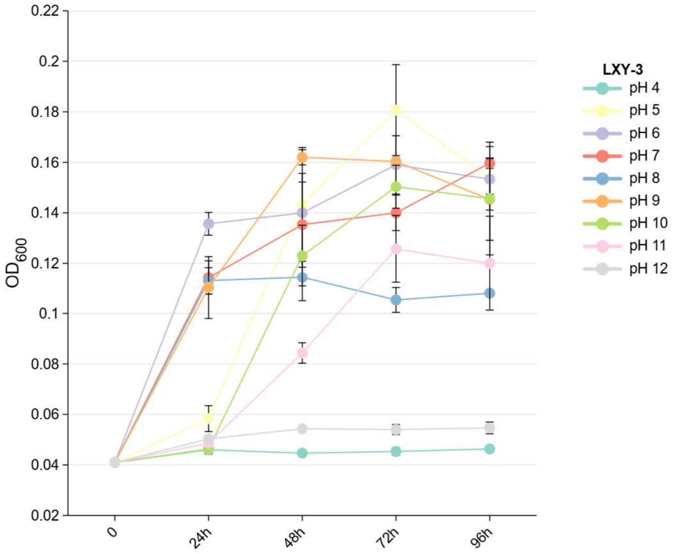

0.22
0.2
0.18
0.16
0.14
0.12
0.1
0.08
0.06
0.04
0.02
OD_{600}
0
24h
48h
72h
96h
LXY-3
pH 4
pH 5
pH 6
pH 7
pH 8
pH 9
pH 10
pH 11
pH 12

(a)

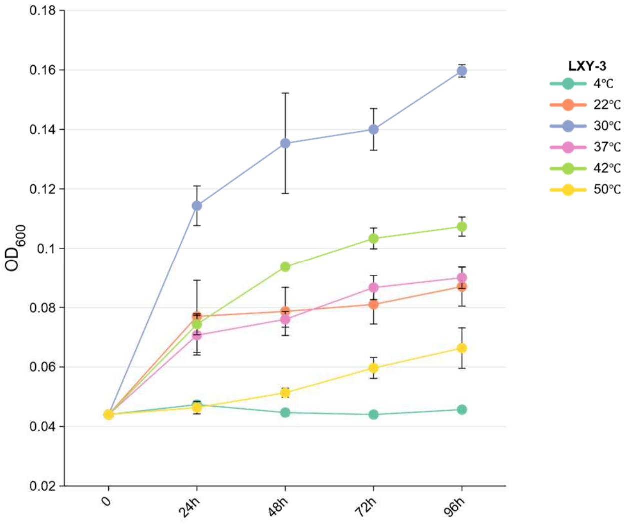

0.18
0.16
0.14
0.12
0.1
0.08
0.06
0.04
0.02
OD_{600}
0
24h
48h
72h
96h
LXY-3
4℃
22℃
30℃
37℃
42℃
50℃

(b)

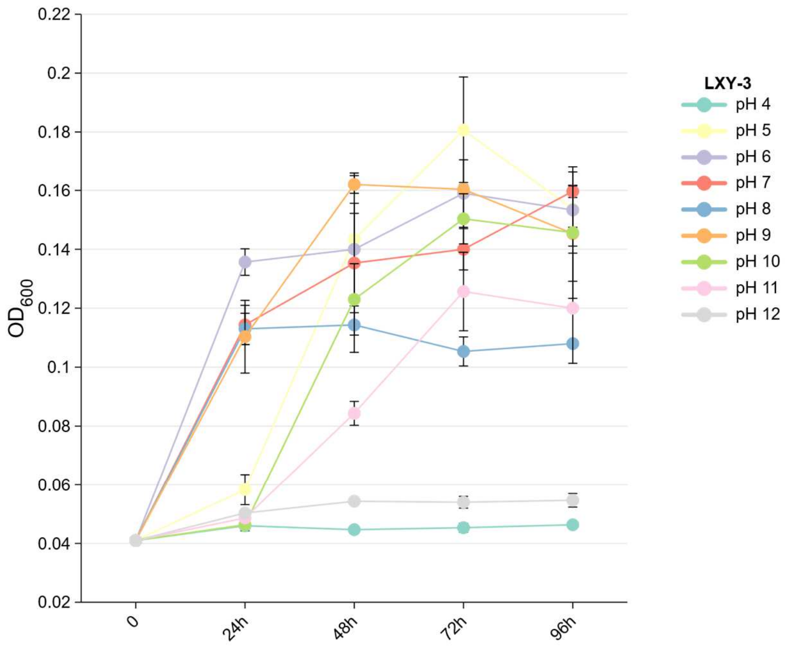

(**c**)

Figure S1. Growth dynamics of strain LXY-3^T under diverse environmental conditions. (**a**) Growth curves (OD_{600}) of LXY-3^T cultured at different pH levels (4–12) at 30°C with 0% NaCl. (**b**) Growth profiles of LXY-3^T across a temperature gradient (4–50°C) at pH 7.0 with 0% NaCl. (**c**) Cell density (OD_{600}) of LXY-3^T in media supplemented with increasing NaCl concentrations (0–5%) at 30°C and pH 7.0.

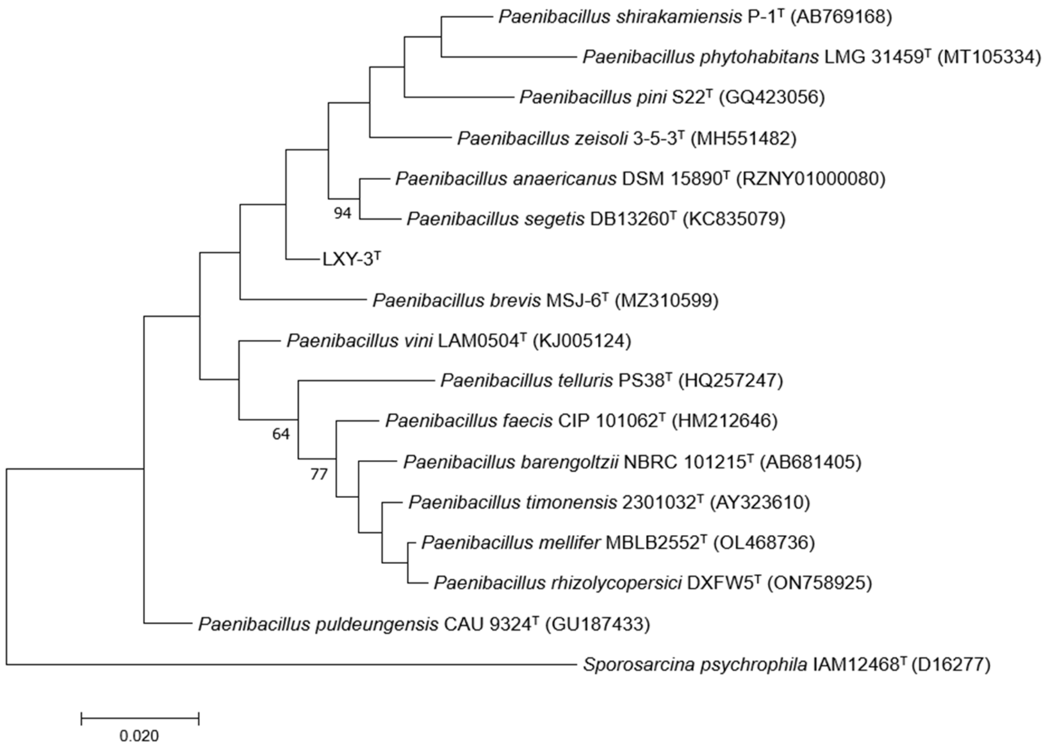

Figure S2. Maximum-likelihood tree based on 16S rRNA gene sequences showing the phylogenetic position of strain LXY-3^{T} among the members of genus *Paenibacillus*. *Sporosarcina psychrophila* IAM12468^{T} served as the out-group. The sequences were obtained from GenBank. Bar, 0.02 substitutions per nucleotide position. The average size of the aligned and trimmed sequences used for phylogenetic tree construction is 1399 bp.

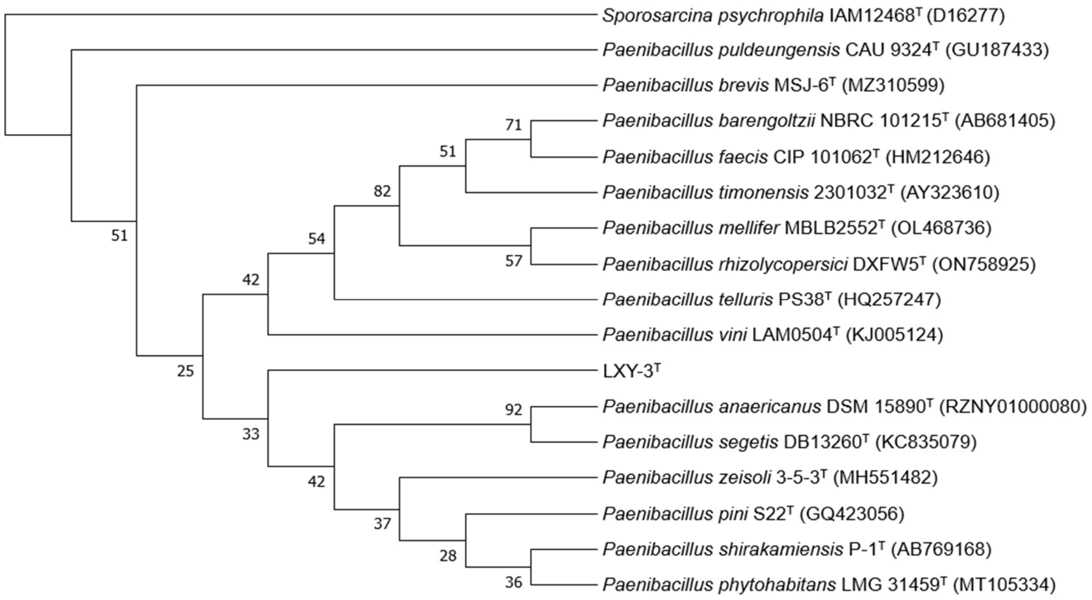

Figure S3. Maximum-parsimony tree based on 16S rRNA gene sequences showing the phylogenetic position of strain LXY-3^{T} among the members of genus *Paenibacillus*. *Sporosarcina psychrophila* IAM12468^{T} served as the out-group. The sequences were obtained from GenBank. The average size of the aligned and trimmed sequences used for phylogenetic tree construction is 1399 bp.

Table S1. Phenotypic characteristics of strain LXY-3^T determined by Biolog GEN III MicroPlate™. "+": Positive; "-": Negative

NO.	Characteristic	LXY-3^T
	Carbon source utilization assay	
1	Negative control	−
A2	Dextrin	+
A3	D-Maltose	+
A4	D-Trehalose	+
A5	D-Cellobiose	+
A6	Gentiobiose	+
A7	Sucrose	−
A8	D-Turanose	+
A9	Stachyose	+
B1	D-Raffinose	−
B2	α-D-Lactose	+
B3	D-Melibiose	+
B4	β-Methyl-D-Glucoside	−
B5	D-Salicin	+
B6	N-Acetyl-D-Glucosamine	+
B7	N-Acetyl-β-D-Mannosamine	+
B8	N-Acetyl-D-Galactosamine	−
B9	N-Acetyl-Neuraminic acid	−
C1	α-D-Glucose	+
C2	D-Mannose	+
C3	D-Fructose	−
C4	D-Galactose	+
C5	3-Methyl glucose	−
C6	D-Fucose	−
C7	L-Fucose	−
C8	L-Rhamnose	−
C9	Inosine	−
D1	D-Sorbitol	−
D2	D-Mannitol	+
D3	D-Arabitol	−
D4	myo-Inositol	−
D5	Glycerol	−
D6	D-Glucose-6-PO4	−
D7	D-Fructose-6-PO4	−
D8	D-Aspartic Acid	−
D9	D-Serine	−
E1	Gelatin	−
E2	Glycyl-L-Proline	−
E3	L-Alanine	−

E4	L-Arginine	–
E5	L-Aspartic acid	–
E6	L-Glutamic acid	–
E7	L-Histidine	–
E8	L-Pyroglutamic acid	–
E9	L-Serine	–
F1	Pectin	–
F2	D-Galacturonic acid	–
F3	L-Galactonic acid lactone	–
F4	D-Gluconic acid	+
F5	D-Glucuronic acid	–
F6	Glucuronamide	–
F7	Mucic acid	–
F8	Quinic acid	–
F9	D-Saccharic acid	–
G1	p-Hydroxy-Phenylacetic acid	–
G2	Methyl pyruvate	–
G3	D-Lactic acid methyl ester	–
G4	L-Lactic acid	–
G5	Citric acid	–
G6	α-Keto-Glutaric acid	–
G7	D-Malic acid	–
G8	L-Malic acid	–
G9	Bromo-Succinic acid	–
H1	Tween 40	–
H2	γ-Amino-Butryric acid	–
H3	α-Hydroxy-Butyric acid	–
H4	β-Hydroxy-D,L-Butyric acid	–
H5	α-Keto-Butyric acid	–
H6	Acetoacetic acid	–
H7	Propionic acid	–
H8	Acetic acid	–
H9	Formic acid	–
	Chemical sensitivity assay	
A10	Positive control	+
A11	pH 6	+
A12	pH 5	+
B10	1% NaCl	–
B11	4% NaCl	–
B12	8% NaCl	–
C10	1% Sodium lactate	–
C11	Fusidic acid	–
C12	D-Serine	–
D10	Troleandomycin	–

D11	Rifamycin SV	–
D12	Minocycline	–
E10	Lincomycin	–
E11	Guanidine HCl	–
E12	Niaproof 4	–
F10	Vancomycin	–
F11	Tetrazolium violet	–
F12	Tetrazolium blue	–
G10	Nalidixic acid	–
G11	Lithium chloride	–
G12	Potassium tellurite	–
H10	Aztreonam	–
H11	Sodium butyrate	–
H12	Sodium bromate	–

Table S2. Phenotypic characteristics of strain LXY-3^T determined by the API ZYM kit. "+": Positive; "-": Negative

NO.	Characteristic	LXY-3^T
1	Control	–
2	Alkaline phosphatase	–
3	Esterase (C4)	+
4	Esterase (C8)	+
5	Lipase (C14)	–
6	Leucine aminopeptidase	–
7	Valine aminopeptidase	–
8	Cysteine aminopeptidase	–
9	Trypsin	–
10	Chymotrypsin	–
11	Acid phosphatase	–
12	Phosphoamidase	+
13	*α*-Galactosidase	+
14	*β*-Galactosidase	+
15	*β*-Glucuronidase	–
16	*α*-Glucosidase	–
17	*β*-Glucosidase	–
18	*N*-acetyl-*β*-glucosaminidase	–
19	α-mannosidase	–
20	α-fucosidase	–

Table S3. Phenotypic characteristics of strain LXY-3^T determined by the API 20NE test kit. "+": Positive; "-": Negative

NO.	Characteristic	LXY-3^T
1	Reduction of nitrates to nitrites	–
2	Reduction of nitrates to nitrogen	–
3	Indole production (tryptophan)	–
4	Fermentation (glucose)	–
5	Arginine dihydrolase	–
6	Urease	–
7	Hydrolysis (β-glucosidase) (esculin)	+
8	Hydrolysis (protease) (gelatin)	–
9	β-Galactosidase	+
10	Assimilation (glucose)	–
11	Assimilation (arabinose)	–
12	Assimilation (mannose)	–
13	Assimilation (mannitol)	–
14	Assimilation (N-acetyl-glucosamine)	–
15	Assimilation (maltose)	–
16	Assimilation (potassium gluconate)	–
17	Assimilation (capric acid)	–
18	Assimilation (adipic acid)	–
19	Assimilation (malate)	–
20	Assimilation (trisodium citrate)	–
21	Assimilation (phenylacetic acid)	–
22	Cytochrome oxidase	+

Table S4. Genome analysis of strain LXY-3^T in KEGG database.

Strain	LXY-1^T
**Celler Processes**	283
1 Cell motility	84
2 Cell growth and death	43
3 Transport and catabolism	15
4 Cellular community-prokaryotes	140
5 Cellular community-eukaryotes	1
**Metabolism**	4294
1 Biosynthesis of other secondary metabolites	106
2 Global and overview maps	2294
3 Xenobiotics biodegradation and metabolism	86
4 Glycan biosynthesis and metabolism	179
5 Carbohydrate metabolism	539
6 Lipid metabolism	142
7 Metabolism of other amino acids	80
8 Amino acid metabolism	290
9 Metabolism of cofactors and vitamins	252
10 Metabolism of terpenoids and polyketides	34
11 Energy metabolism	164
12 Nucleotide metabolism	128
**Genetic Information Processing**	275
1 Translation	99

2 Replication and repair	111
3 Folding, sorting and degradation	55
4 Transcription	10
**Human Diseases**	222
1 Endocrine and metabolic disease	8
2 Infectious disease: bacterial	41
3 Cancer: overview	40
4 Neurodegenerative disease	20
5 Cardiovascular disease	24
6 Cancer: specific types	3
7 Infectious disease: parasitic	5
8 Drug resistance: antineoplastic	7
9 Infectious disease: viral	6
10 Drug resistance: antimicrobial	67
11 Immune disease	1
**Environmental Information Processing**	616
1 Signaling molecules and interaction	1
2 Signal transduction	314
3 Membrane transport	301
